# Prognostic significance of age in 5631 patients with Wilms tumour prospectively registered in International Society of Paediatric Oncology (SIOP) 93-01 and 2001

**DOI:** 10.1371/journal.pone.0221373

**Published:** 2019-08-19

**Authors:** J. A. Hol, M. I. Lopez-Yurda, H. Van Tinteren, M. Van Grotel, J. Godzinski, G. Vujanic, F. Oldenburger, B. De Camargo, G. L. Ramírez-Villar, C. Bergeron, K. Pritchard-Jones, N. Graf, M. M. Van den Heuvel-Eibrink

**Affiliations:** 1 Princess Máxima Center for Pediatric Oncology, Utrecht, The Netherlands; 2 Department of Biometrics, Netherlands Cancer Institute, Amsterdam, The Netherlands; 3 Department of Paediatric Surgery, Marciniak Hospital, Wroclaw, Poland; 4 Department of Paediatric Traumatology and Emergency Medicine, Medical University, Wroclaw, Poland; 5 Department of Pathology, Sidra Medicine, Doha, Qatar; 6 Department of Radiotherapy, Academic Medical Center, Amsterdam, The Netherlands; 7 Paediatric Haematology-Oncology Program, Instituto Nacional de Cancer (INCA), Rio de Janeiro, Brazil; 8 Department of Paediatric Oncology, Hospital Universitario Virgen del Rocío, Seville, Spain; 9 Department of Paediatric Oncology, Institut d'Hematologie et d'Oncologie Pédiatrique, Centre Léon Bérard, Lyon, France; 10 UCL Great Ormond Street Institute of Child Health, University College London, London, United Kingdom; 11 Department of Paediatric Oncology & Haematology, Saarland University, Homburg, Germany; Universidad de Navarra, SPAIN

## Abstract

**Background:**

To enhance risk stratification for Wilms tumour (WT) in a pre-operative chemotherapy setting, we explored the prognostic significance and optimal age cutoffs in patients treated according to International Society of Paediatric Oncology Renal Tumour Study Group (SIOP-RTSG) protocols.

**Methods:**

Patients(6 months-18 years) with unilateral WT were selected from prospective SIOP 93–01 and 2001 studies(1993–2016). Martingale residual analysis was used to explore optimal age cutoffs. Outcome according to age was analyzed by uni- and multivariable analysis, adjusted for sex, biopsy(yes/no), stage, histology and tumour volume at surgery.

**Results:**

5631 patients were included; median age was 3.4 years(IQR: 2–5.1). Estimated 5-year event-free survival (EFS) and overall survival (OS) were 85%(95%CI 83.5–85.5) and 93%(95%CI 92.0–93.4). Martingale residual plots detected no optimal age cutoffs. Multivariable analysis showed lower EFS with increasing age(linear trend *P<*0.001). Using previously described age categories, EFS was lower for patients aged 2-4(HR 1.34, *P =* 0.02), 4-10(HR 1.83, *P<*0.0001) and 10–18 years(HR 1.74, *P =* 0.01) as compared to patients aged 6 months-2 years. OS was lower for patients 4–10 years(HR 1.67, *P =* 0.01) and 10–18 years(HR 1.87, *P =* 0.04), but not for 2–4 years(HR 1.29, *P =* 0.23). Higher stage, histological risk group and tumour volume were independent adverse prognostic factors.

**Conclusion:**

Although optimal age cutoffs could not be identified, we demonstrated the prognostic significance of age as well as previously described cutoffs for EFS (2 and 4 years) and OS (4 years) in children with WT treated with pre-operative chemotherapy. These findings encourage the consideration of age in the design of future SIOP-RTSG protocols.

## Introduction

As treatment for Wilms tumour (WT) is evolving towards further risk adaptation, there is an increasing interest in additional factors that can help to stratify treatment intensity based on the patient’s individual risk. One of these factors appears to be a patient’s age at diagnosis. Older age has been suggested to be an adverse prognostic factor for recurrence and mortality[1–4] while younger patients may need less intensive treatment.[5–8]

Treatment stratification of WT has been primarily based on pathological stage and histology. More recently, potential molecular prognostic markers such as copy number changes and loss of heterozygosity (LOH) of specific chromosomal regions are emerging.[9–15] Currently, in addition to tumour weight, LOH 1p/16q, stage and histology, the Children’s Oncology Group (COG) includes age in the risk stratification of its most recent protocols.[5, 7] So far, the independent prognostic significance of age has not been sufficiently validated in a large cohort of patients treated with pre-operative chemotherapy, as recommended in International Society of Paediatric Oncology Renal Tumour Study Group (SIOP-RTSG) protocols (**S1 Table**).

Age as a prognostic factor was first described in 1976 when D’Angio et al.[1] reported that the addition of postoperative radiation therapy did not improve the already excellent outcomes of patients <2 years with stage I disease, treated with primary surgery in National Wilms tumour Study (NWTS)-1.[1] After a pooled analysis of NWTS-1, -2 and -3, the ‘very low risk’ patients <2 years with stage I, non-anaplastic WT(lymph node sampling required), tumour weight <550 grams, without predisposition syndromes, were subsequently treated with nephrectomy only, in Children’s Oncology Group (COG) protocols.[5–8]

Whether the age of 2 years is the optimal cutoff for risk stratification was debated in a later study by the UK Children’s Cancer Study Group, suggesting that the age of 4 years may be a more relevant cutoff in the setting of minimal adjuvant chemotherapy.[3] The Associazione Italiana Ematologia Oncologia Pediatrica (AIEOP) study that used a cutoff at the age of 2 years, did not find older age to be an independent prognostic factor in stage I-IV WT.[16] By contrast, the relatively small subgroup of WT patients older than 10 years, revealed a particularly poor survival(63–70%) in reports from the Automated Childhood Cancer Information System (ACCIS) and UK Children’s Cancer And Leukemia Group, compared to a survival of 80–90% in younger patients.[17, 18]

In the current study, we aimed to assess the prognostic significance of age in a large, prospectively registered cohort of paediatric patients with WT treated with pre-operative chemotherapy according to recent SIOP protocols. Moreover, we aimed to identify relevant age cutoffs for future stratification purposes.

## Patients and methods

### Patients

Patients with histologically proven stage I-IV WT, aged 6 months-18 years, treated according to SIOP 93–01[19] and SIOP 2001[20] protocols (including the SIOP WT 2001 trial with EudraCT number 2007-004591-39) from 1993–2016 and prospectively registered in the SIOP database, were included in this retrospective analysis. The SIOP-RTSG steering committee approved the research proposal for this specific study and anonymized data were made available to the researchers through statistical reports generated by data scientists of the SIOP-RTSG office. Patients <6 months were excluded as they received separate treatment regimen.[21] Moreover, patients with bilateral disease, non-Wilms tumours or extrarenal tumour sites were excluded. Subsets of patients from the SIOP database had been previously described in several reports.[9, 18–20, 22, 23] For both protocols ethical approval was obtained by ethical committees of all participating countries, and written informed consent for participation was obtained from the parents or legal representatives of the patients.

### SIOP 93–01 and SIOP 2001 protocols

Pre-operative chemotherapy consisted of 4 weeks of vincristine and actinomycin-D in case of localized disease, and 6 weeks of vincristine, actinomycin-D and doxorubicin in case of metastatic disease. Biopsy before start of treatment was not recommended as a standard procedure, but was allowed without upstaging if performed by a percutaneous fine needle or trucut procedure. This was a routine procedure in the Children’s Cancer and Leukemia Group (CCLG), including the UK and Republic of Ireland, that participated in SIOP 2001 but not SIOP 93–01. Post-operative treatment stratification depended on SIOP stage and histological risk group [24, 25], and evolved over time. In SIOP 93–01, post-operative chemotherapy was randomized for stage I intermediate-risk and anaplastic WT, with the trial arm receiving a shorter treatment regimen, which was subsequently adopted for intermediate-risk WT in SIOP 2001.[19] Non-viable tumour tissue in the renal sinus and perirenal fat was no longer taken into account for upstaging histological risk group in SIOP 2001. Moreover, focal anaplasia (which was considered high-risk in SIOP 93–01) was considered intermediate risk and treated accordingly; while blastemal-type WT was considered high-risk and treated accordingly.[23, 24] For stage II-III intermediate risk tumours, the SIOP 2001 randomized trial provided evidence for omitting doxorubicin, and this was adjusted accordingly from 2011 onwards in the continuation of the SIOP 2001 protocol.[20]

In the current analysis, high-risk tumours included diffuse anaplastic and/or blastemal-type WT after pre-operative chemotherapy. Intermediate risk tumours were either stromal, epithelial, mixed or regressive type or focal anaplasia, while low risk was defined as completely necrotic tumours after pre-operative chemotherapy. Central pathology review was performed for 83.4% of patients in SIOP 2001, and for 94.4% of patients in SIOP 93–01. Tumour volume was not a factor for treatment stratification, with the exception of German Paediatric Oncology Haematology (GPOH) centers, where patients with non-stromal, non-epithelial intermediate risk WT and a tumour volume >500mL at surgery, received ‘high-risk’ post-operative chemotherapy (four drugs).[26]

### Statistical methods

To search for potential relevant prognostic age cutoffs in relation to event-free survival (EFS) and overall survival (OS), martingale residual plots were evaluated.[27] Cox regression models were used to analyze the prognostic significance of age. Variables assessed in univariable analysis were age, sex, biopsy (yes/no), overall stage, histological classification and tumour volume at surgery(radiologically assessed, dichotomized as ≤500 ml and >500 ml). Variables that appeared to be associated with EFS/OS in univariable analysis (*P<*0.10) and/or were known confounders based on previous literature, were entered into the multivariable model, stratified by national/regional group and study protocol (SIOP 2001 and SIOP 93–01). Patient characteristics were compared using Pearson’s Chi-Squared test for categorical variables, and Mann Whitney’s U-test/Kruskall-Wallis for continuous variables.

Tumour volume at surgery was missing in 18·3% of cases, and was imputed using multiple imputation techniques (fully conditional method) on 100 generated datasets, assuming it was missing at random. Missing volume was associated with center/consortium and not with patient characteristics, and there were no indications that it might be related to unobserved characteristics or the missing volume itself. Resulting model estimates were combined using SAS PROC MIANALYZE software (version 9.4).

## Results

### Patient characteristics

**Fig 1:**
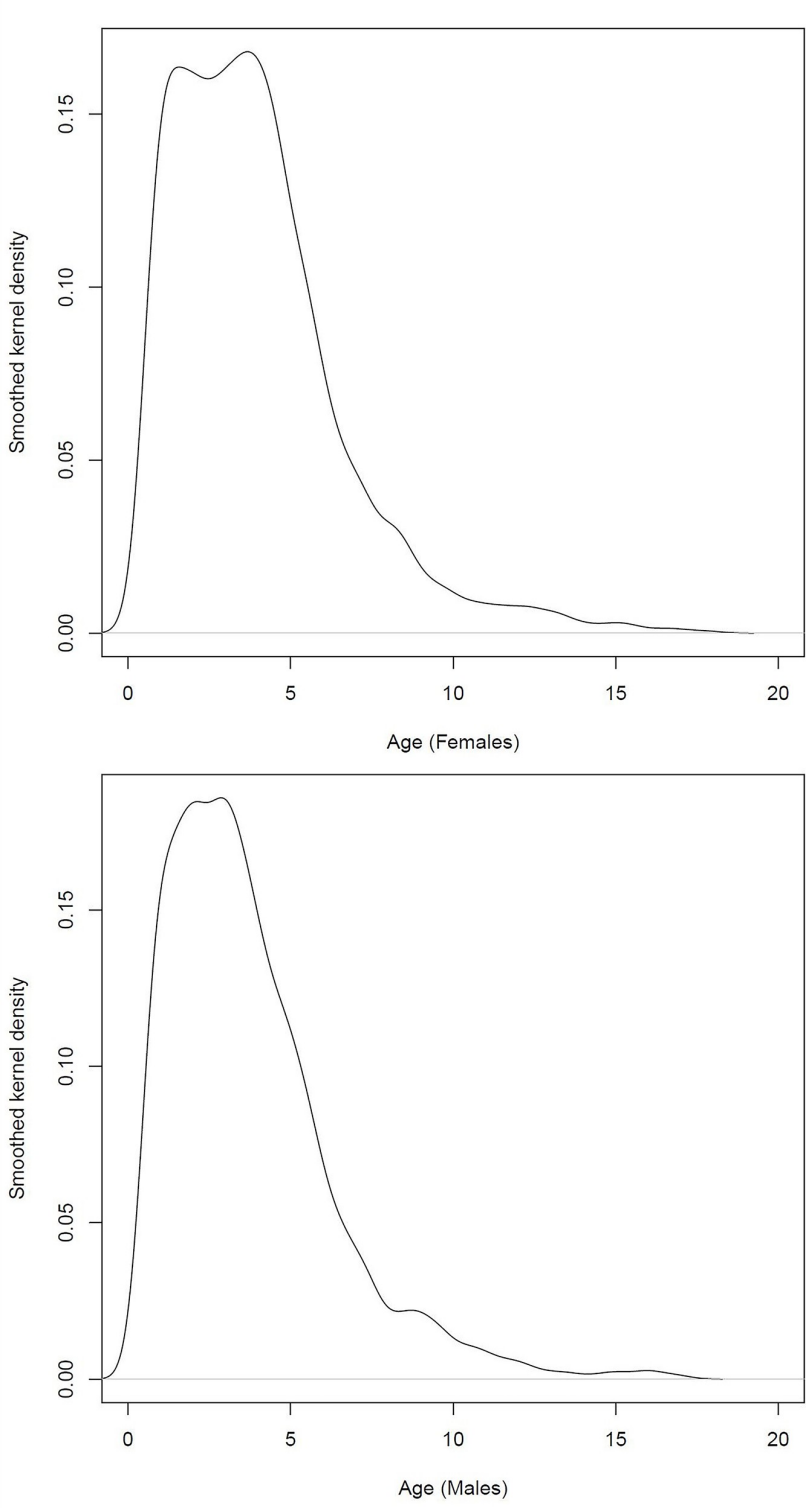
**Distribution of age at diagnosis (smoothed kernel density), displayed for females (top) and males (bottom).** Median age was 3·6 years (IQR 2–5·2) for females and 3·2 years (IQR 1·9–4·9) for males (*P<*0.001).

Out of 7262 registered patients with histologically proven WT, 5631 met the inclusion criteria (78%) (**S1 Fig**). Baseline characteristics are summarized in **Table 1**. Median follow-up at time of data capture was 6·3 years (interquartile range, IQR: 3·0–8·7). Median age at diagnosis was 3·4 years (IQR: 2–5·1) with only 189 patients (3·4%) aged 10 years or older. Median age was 3·6 (IQR 2–5·2) for females and 3·2 (IQR 1·9–4·9) for males (*P<*0.001). The age distribution of females showed two peaks, at around 1 year and 4 years. For males there was an early peak in the age distribution but bimodality was less apparent than for females (**Fig 1**). Forty-five percent (*N =* 2554) presented with overall stage I disease, 23% (*N =* 1271) with stage II, 17% (*N =* 949) with stage III and 15% (*N =* 857) with stage IV. WT’s were histologically classified as low-risk in 5·6% (*N =* 315), intermediate risk in 82% (*N =* 4566), high-risk blastemal type in 8·3% (*N =* 466) and high-risk diffuse anaplastic in 4·9% (*N =* 278). Biopsies were performed in 208 cases (10·5%) in SIOP 93–01 and 1159 cases (31·7%) in SIOP 2001. Tumour volume at surgery was available for 4599 patients (81.7%), of whom 14·1% (*N =* 649) had a tumour volume of >500ml at surgery.

**Table 1:** Baseline patient and disease characteristics (*N* = 5631).

		TOTAL	
		*N*	%
**Protocol**	SIOP 93–01	**1980**	***35·2***
	SIOP 2001	**3651**	***64·8***
**Sex**	Female	3023	*53·7*
	Male	2608	*46·3*
	*Missing*	0	*0*
**Age**	6 mths– 2 yrs	1439	*25·6*
	2–4 yrs	1939	*34·4*
	4–10 yrs	2064	*36·7*
	10–18 yrs	189	*3·4*
	*Missing*	0	*0*
**Histology**	Low risk	315	*5·6*
	Intermediate risk	4566	*81·1*
	High risk–blastemal type	466	*8·3*
	High risk–diffuse anaplastic	278	*4·9*
	*Missing*	6	*0·1*
**SIOP overall stage**	I	2554	*45·5*
	II	1271	*22·6*
	III	949	*16·9*
	IV	857	*15·2*
	*Missing*	0	*0*
**SIOP abdominal stage**	I	2766	*49·3*
	II	1491	*26·6*
	III	1354	*24·1*
	*Missing*	4	*0·1*
**Side**	Left	2880	*51·2*
	Right	2749	*48·8*
	*Missing*	2	*0·03*
**Biopsy**	Yes	1367	*24·3*
	No	4264	*75·7*
	*Missing*	0	*0*
**Volume at surgery**	≤500 ml	3950	*70·1*
	>500 ml	649	*11·5*
	*Missing*	1032	*18·3*

### Comparison of patient characteristics between age groups

The distribution of stage, histological risk and tumour volume differed between age groups, with the frequency of metastatic disease, high-risk histology types (most markedly for diffuse anaplastic WT) and high-volume tumours increasing with age (**Table 2**).

**Table 2:** Comparison of patient characteristics between age groups (SIOP 93–01 and SIOP 2001).

		0–2 years		2–4 years		4–10 years		10–18 years		Total		*p-value*
		N	%	N	%	N	%	N	%	N	%	
**SIOP stage**	Stage I	938	*65·2*	841	*43·3*	722	*35·0*	53	*28·0*	2554	*45·4*	<0·001
	Stage II	272	*18·9*	471	*24·3*	479	*23·2*	49	*25·9*	1271	*22·6*	
	Stage III	163	*11·3*	332	*17·1*	416	*20·2*	38	*20·1*	949	*16·9*	
	Stage IV	66	*4·6*	295	*15·2*	447	*21·7*	49	*25·9*	857	*15·2*	
**Histology**	Low risk	60	*4·2*	77	*4*	161	*7·8*	17	*9*	315	*5·6*	<0·001
	Intermediate risk	1261	*87·8*	1637	*84·5*	1533	*74·3*	135	*71·8*	4566	*81·2*	
	High risk: diffuse anaplastic	12	*0·8*	81	*4·2*	175	*8·5*	10	*5·3*	278	*4·9*	
	High risk: blastemal type	105	*7·3*	142	*7·3*	193	*9·4*	26	*13·8*	466	*8·3*	
**Volume at surgery**	≤500 ml	980	*83·2*	1411	*88·4*	1462	*86·9*	97	*68·3*	3950	*85·9*	<0·001
	>500 ml	198	*16·8*	185	*11·6*	221	*13·1*	45	*31·7*	649	*14·1*	

### Optimal age cutoffs

**Fig 2:**
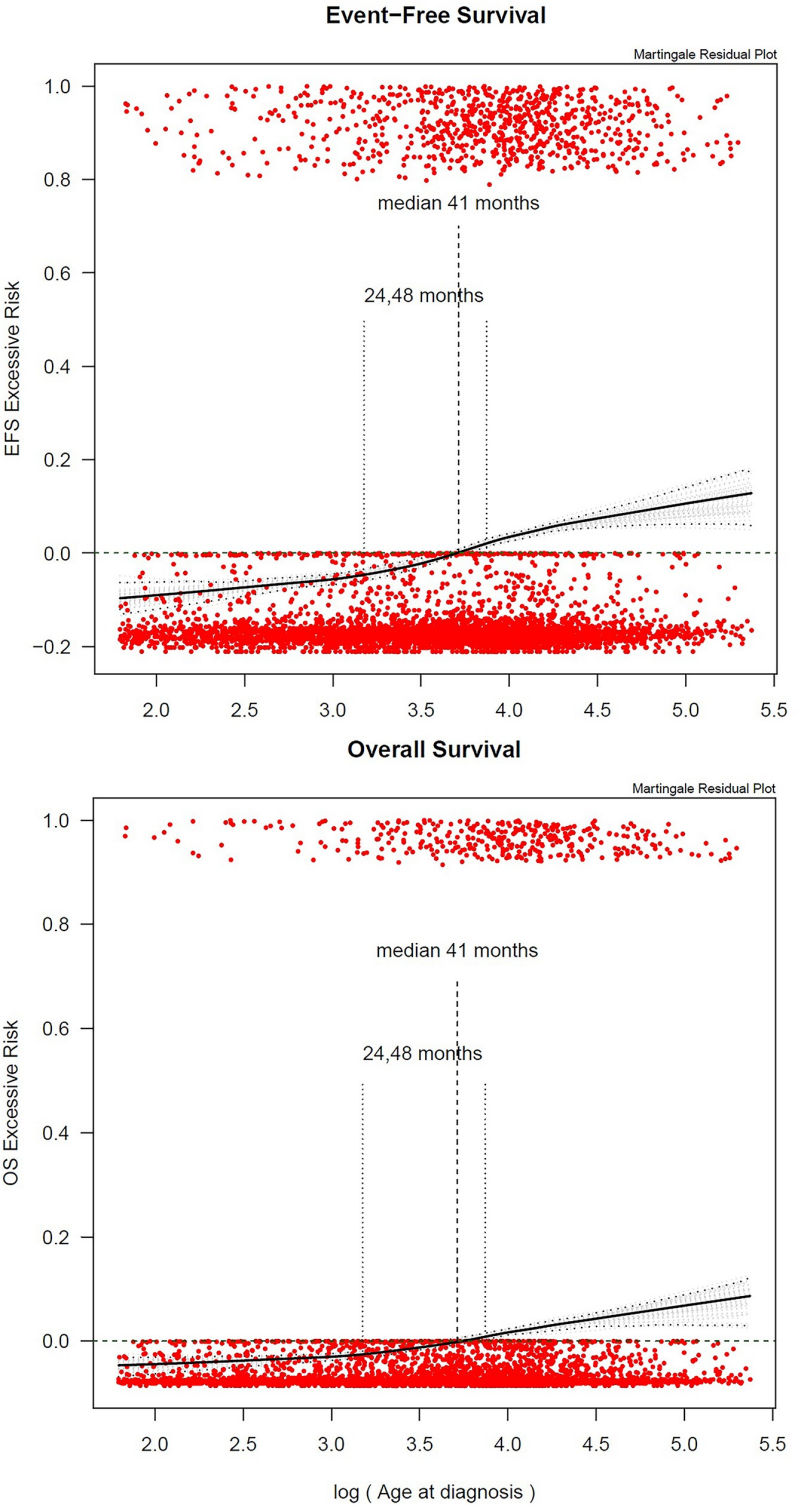
**Martingale residual plots showing excessive risk for EFS (top) and OS (bottom) plotted versus log (age).** The vertical axis in these plots can be interpreted as excess risk (increasing from bottom to top) and the horizontal axis is age (logarithmic scale). A smoothed curve (LOWESS: locally weighted scatterplot smoothing) is displayed for assessing the functional form for age. Median age (of the log or ratio) is indicated in the martingale residual plots with a dotted line. The grey lines in the plots correspond to the 95% bootstrapped confidence interval. The plots suggest that an increase in age is linearly associated with the risk of an event. No specific change point (knot) can be clearly observed.

Martingale residual plots (**Fig 2**) suggested that an increase in age was linearly associated with the risk of an event. However, since no specific change point (knot) could be clearly observed, hence, no optimal cutoffs for categorizing age could be identified. Therefore, in further analyses, age was included as a linear factor (per year), as well as categorized according to previous studies at the ages of 2, 4 and 10 years.

### Survival and univariable analysis of prognostic factors

**Fig 3:**
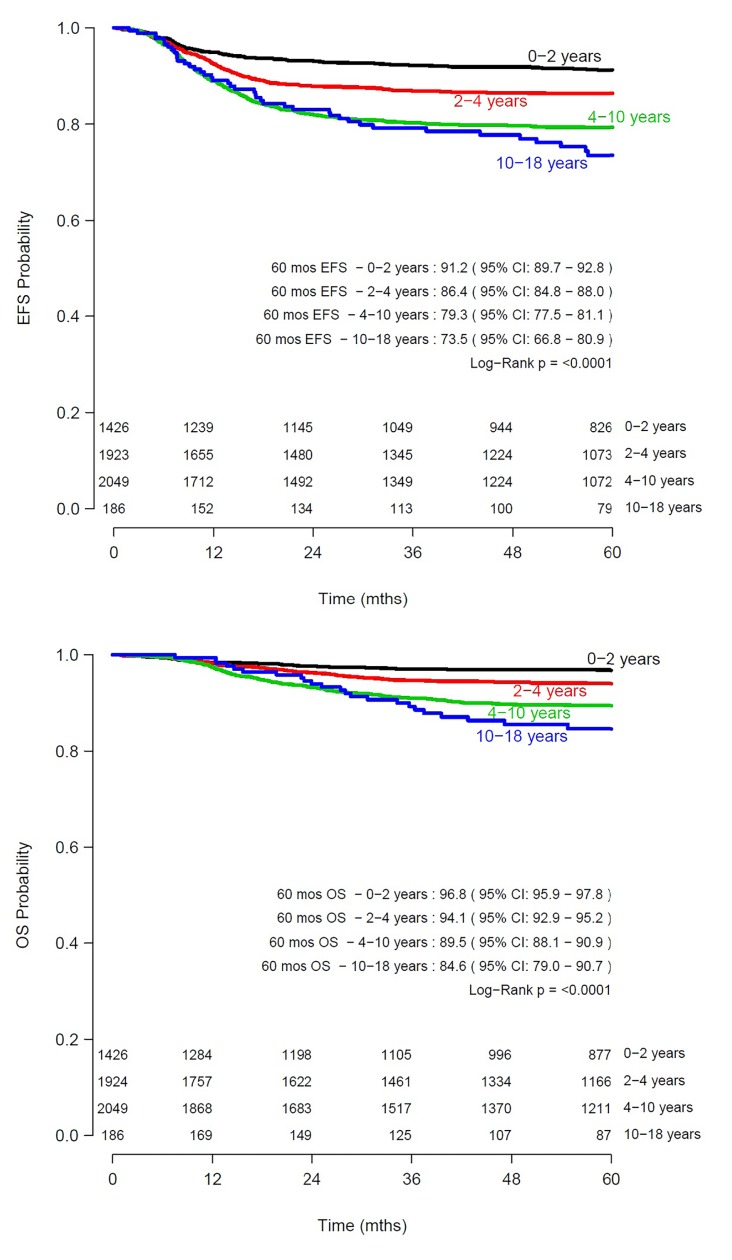
Survival of paediatric patients with Wilms tumour according to age. Kaplan Meier curves showing estimated 5-year event-free survival (EFS) (top) and estimated 5-year overall survival (OS) (bottom) per age category, N = 5585.

5585 patients were included in the survival analysis, after censoring 46 patients without available follow-up data. Estimated 5-year EFS and OS of the total cohort was 85% (95% CI 83·5–85·5) and 93% (95% CI 92·0–93·4) respectively. A total of 836 events occurred, of which 93.8% (*N =* 784) were relapses. In univariable analysis, significant differences in EFS and OS were found between age categories 6 months– 2 years, 2–4, 4–10 and ≥10 years (**Fig 3**). The 5-year EFS was 91·2% (95% CI 89·7–92·8) for ages 6 months-2 years, 86·3% (95% CI 84·7–87·9) for 2–4 years, 79·3% (95% CI 77·5–81·1) for 4–10 years and 73·5% (95% CI 66·8–80·9) for 10–18 years (log rank *P<*0·0001). OS was 96·8% (95% CI 95·9–97·8) for ages 6 months-2 years, 94·1% (95% CI 92·9–95·2) for 2–4 years, 89·5% 95% CI 88·1–90·9) for 4–10 years and 84·6% (95% CI 79·0–90·7) for 10–18 years (*P<*0·0001).

### Multivariable analysis of prognostic factors for EFS

Age categorized as 6 months– 2 years, 2–4 years, 4–10 years and 10–18 years, was a significant prognostic factor for EFS in multivariable analysis (2–4 years: adjusted HR 1·34, *P =* 0·02, 4–10 years: adjusted HR 1·83, *P<*0·0001, 10–18 years: adjusted HR 1·74, *P =* 0·01), after stratifying for national/regional study group and study protocol, and including sex, overall stage, histological risk group, biopsy and tumour volume at surgery. Other independent prognostic factors for EFS were overall stage III and IV, histological subtype (low, intermediate or high-risk) and tumour volume at surgery (**Table 3**).When replacing the age categories by age per year in the multivariable model, the linear trend observed in the Martingale residual plot was confirmed for EFS (adjusted HR 1·06, *P<*0·0001). The same conclusions were obtained when imputing missing volume (**S2 Table**), and when limiting the analysis to SIOP 2001 patients only (N = 3132, **S3 Table**).

**Table 3:** Prognostic factors for event-free survival (EFS) in patients with Wilms tumour (*N =* 4596).

Characteristic		Events	Univariable		Multivariable, age categorized		Multivariable, age linear	
			HR (95% CI)	p-value	HR (95% CI)	p-value	HR (95% CI)	p-value
**Sex**	Female	361	1		1		1	
	Male	305	0·98 (0·84–1·14)	0·78	1 (0·86–1·17)	1	0·99 (0·85–1·15)	0·88
**Age at diagnosis, categorized (years)**	0–2	97	1		1			
	2–4	203	1·56 (1·23–1·99)	0·0003	1·34 (1·05–1·72)	0·02		
	4–10	332	2·49 (1·99–3·12)	< 0·0001	1·83 (1·44–2·32)	< 0·0001		
	10–18	34	3·18 (2·15–4·70)	< 0·0001	1·74 (1·15–2·61)	0·01		
**Age at diagnosis,** **linear (years)**		666	1·12 (1·09–1·15)	< 0·0001			1·06 (1·03–1·09)	< 0·0001
**Overall stage**	I	204	1		1		1	
	II	134	1·30 (1·04–1·61)	0·019	1·13 (0·91–1·41)	0·28	1·17 (0·94–1·46)	0·17
	III	146	2·07 (1·68–2·56)	< 0·0001	1·6 (1·28–2)	< 0·0001	1·66 (1·33–2·07)	< 0·0001
	IV	182	3·08 (2·52–3·77)	< 0·0001	2·97 (2·4–3·67)	< 0·0001	3·13 (2·54–3·86)	< 0·0001
**Histological risk group**	Intermediate risk	471	1		1		1	
	High risk: diffuse Anaplastic	92	14·71 (12·44–17·38)	< 0·0001	2·9 (2·29–3·68)	< 0·0001	3·12 (2·48–3·94)	< 0·0001
	High risk: blastemal type	90	2·48 (1·94–3·17)	< 0·0001	2·16 (1·72–2·72)	< 0·0001	2·13 (1·69–2·69)	< 0·0001
	Low risk	13	0·59 (0·35–1·01)	0·052	0·27 (0·15–0·46)	< 0·0001	0·28 (0·16–0·48)	< 0·0001
**Biopsy**	No	463	1		1		1	
	Yes	203	1·44 (1·22–1·70)	< 0·0001	1·1 (0·89–1·37)	0·37	1·06 (0·85–1·31)	0·61
**Volume at surgery**	≤500 ml	502	1		1		1	
	>500 ml	164	2·24 (1·88–2·68)	< 0·0001	2·03 (1·69–2·44)	< 0·0001	1·93 (1·6–2·32)	< 0·0001

Univariable and Multivariable Cox regression models of event-free survival (EFS), stratified by national/regional study group and database (SIOP 93–01 and SIOP 2001), with age categorized and age linear.

### Multivariable analysis of prognostic factors for OS

For OS, the age category 2–4 years (adjusted HR 1·23, *P =* 0·29) did not retain significance in multivariable analysis. However, patients aged 4–10 (adjusted HR 1·67, *P =* 0·01) and 10–18 years (adjusted HR 1·87, *P =* 0·04) revealed lower OS compared to patients aged 6 months– 2 years. Other factors that were significantly associated with OS included overall stage III and IV, histological classification and tumour volume at surgery (**Table 4**). When including age as a continuous variable in the multivariable model, this did not reach statistical significance (adjusted HR 1·04, *P =* 0·06). These conclusions were maintained when imputing missing volume (**S4 Table**). When limiting the analysis to SIOP 2001 patients, age (categorized or continuous) did not retain significance (N = 3132, **S5 Table**).

**Table 4:** Prognostic factors for overall survival (OS) in patients with Wilms tumour (*N =* 4596).

Characteristic		Events	Univariable		Multivariable, age categorized		Multivariable, age linear	
			HR (95% CI)	p-value	HR (95% CI)	p-value	HR (95% CI)	p-value
**Sex**	Female	168	1		1		1	
	Male	123	0·84 (0·67–1·06)	0·14	0·85 (0·67–1·07)	0·16	0·84 (0·66–1·06)	0·13
**Age at diagnosis, categorized (years)**	0–2	31	1		1			
	2–4	83	1·96 (1·30–2·96)	0·0014	1·29 (0·85–1·97)	0·23		
	4–10	159	3·63 (2·47–5·33)	< 0·0001	1·67 (1·11–2·51)	0·01		
	10–18	18	5·16 (2·89–9·23)	< 0·0001	1·87 (1·02–3·44)	0·04		
**Age at diagnosis,** **linear (years)**		291	1·15 (1·11–1·19)	< 0·0001			1·04 (1–1·08)	0·06
**Overall stage**	I	56	1		1		1	
	II	48	1·69 (1·15–2·49)	0·0076	1·43 (0·97–2·11)	0·07	1·47 (1–2·17)	0·05
	III	76	3·91 (2·77–5·52)	< 0·0001	2·76 (1·93–3·94)	< 0·0001	2·86 (2·01–4·09)	< 0·0001
	IV	111	6·65 (4·82–9·17)	< 0·0001	6·78 (4·82–9·53)	< 0·0001	7·14 (5·09–10·01)	< 0·0001
**Histological risk group**	Intermediate risk	152	1		1		1	
	High risk: diffuse Anaplastic	73	10·13 (7·66–13·40)	< 0·0001	6·91 (5·09–9·39)	< 0·0001	7·38 (5·47–9·95)	< 0·0001
	High risk: blastemal type	57	4·01 (2·96–5·44)	< 0·0001	4·55 (3·31–6·25)	< 0·0001	4·58 (3·33–6·3)	< 0·0001
	Low risk	9	0·86 (0·44–1·68)	0·65	0·53 (0·27–1·05)	0·07	0·55 (0·28–1·09)	0·09
**Biopsy**	No	192	1		1		1	
	Yes	99	1·66 (1·30–2·11)	< 0·0001	1·03 (0·75–1·4)	0·87	1·03 (0·75–1·41)	0·85
**Volume at surgery**	≤500 ml	201	1		1		1	
	>500 ml	90	2·99 (2·33–3·83)	< 0·0001	2·23 (1·7–2·91)	< 0·0001	2·19 (1·67–2·85)	< 0·0001

Univariable and Multivariable Cox regression models of overall survival (OS), stratified by national/regional study group and database (SIOP 93–01 and SIOP 2001), with age categorized and age linear.

## Discussion

This study, which included 5631 patients with unilateral WT registered over 23 years in the recent SIOP trials, demonstrated that age is an independent prognostic factor for EFS in patients treated with pre-operative chemotherapy. Although optimal age cutoffs for risk stratification could not be identified, the prognostic significance of previously described cutoffs was confirmed for EFS (2 and 4 years) and OS (4 years). Despite the observation that older patients more frequently have a higher stage at diagnosis, high-risk histology types and large-volume tumours, age retained independent prognostic significance. Interestingly, the strong prognostic value of tumour volume ≥500mL confirmed previous findings from the posthoc analysis of the SIOP 2001 randomized trial.[20, 26] While previous studies have reported conflicting results on the prognostic value of age, depending on sample size and whether age was included as a categorized or continuous variable, our findings are in line with the results described in other large cohorts (>1000 patients, **S1 Table**).

We observed that the presence of diffuse anaplasia increases with age, and is a strong adverse prognostic factor. We could not include molecular markers in the analysis, since copy number status was only available for a subset of the SIOP 2001 cohort (N = 586), as previously described.[9] Gain of 1q and loss/LOH of 1p/16q, which are thought to reflect genomic instability, have been associated with adverse outcome in various reports.[9–15] These and other copy number changes/LOH appear to be more prevalent in older patients.[11, 12, 15] Three recent studies that assessed age, 1q gain and 1p/16q loss/LOH in multivariable analysis[9, 13, 14] found 1q gain to be independently associated with relapse and/or survival, while age and 1p/16q loss/LOH did not retain significance (**S6 Table**). A large study on prognostic molecular markers (*N =* 1114) showed that 1p/16q loss/LOH was not independently associated with EFS when correcting for 1q gain, but suggested prognostic value in the group of patients lacking 1q gain.[11] Age and 1q gain have not been combined in multivariable models with >1000 patients, but will be prospectively validated in the UMBRELLA SIOP-RTSG protocol.[26, 28, 29] Noteworthy, different biomarkers may be important in patients aged <2 years, particularly in a nephrectomy-only setting, where 11p15 status was shown to be associated with relapse.[7, 30]

Furthermore, Wilms tumour predisposition syndromes may be a relevant factor to consider in relation to age at diagnosis and survival, but could not be assessed in this study due to incomplete data. Wilms tumour predisposition syndromes have been associated with a younger age at diagnosis and depending on the genetic aberration, a more favorable tumour biology. On the other hand, these syndromes carry a higher risk of bilateral/second tumours and subsequent renal failure. Wilms tumour predisposition was not always evaluated or recognized in the past, and incompletely registered, as this was beyond the objectives of SIOP 93–01 and 2001. Therefore, we were unable to reliably distinguish between patients with and without a Wilms tumour predisposition syndrome in the current study. As genomic sequencing becomes more widely implemented in paediatric oncology, more data will become available to unravel these associations in the SIOP-RTSG UMBRELLA protocol.[26]

Other limitations of this study included the long period of time during which treatment evolved based on the results of two successive clinical trials, and missing data requiring imputation. When limiting the analysis to SIOP 2001 only, a more uniform but slightly smaller cohort, age retained significance in relation to EFS but not OS.

The two most recent COG protocols have provided some insight into the outcomes after reduced treatment for young patients[5, 7], but this is difficult to compare to SIOP-RTSG protocols, in which response to pre-operative chemotherapy influences risk stratification.[24] Yet, since age seems to emerge as an even more important adverse prognostic factor in reduced therapy settings[3], it seems sensible to remove older patients from minimal treatment strategies. A decision analysis approach, simulating reduced treatment to model the clinical course in different age categories, could aid the design of future guidelines for treatment stratification.[31]

Overall, these results encourage the consideration of age in the design of future SIOP-RTSG protocols, albeit after validation of 1q gain, other molecular markers and age as independent prognostic factors in the UMBRELLA SIOP-RTSG protocol.

## Supporting information

S1 Fig Inclusion flowchart of patients with histologically proven Wilms tumour from the SIOP 93–01 and SIOP 2001 database. (DOCX)

S1 Table Previously published literature including age as a variable for outcome in Wilms tumour (WT). (DOCX)

S2 Table Prognostic factors for event-free survival (EFS) in patients with Wilms tumour, missing volume imputed (N = 5631). (DOCX)

S3 Table Prognostic factors for event-free survival (EFS) in patients with Wilms tumour, SIOP 2001 only (N = 3132). (DOCX)

S4 Table Prognostic factors for overall survival (OS) in patients with Wilms tumour, missing volume imputed (N = 5631). (DOCX)

S5 Table Prognostic factors for overall survival (OS) in patients with Wilms tumour, SIOP 2001 only (N = 3132). (DOCX)

S6 Table Previously published studies including 1q gain in multivariable analysis. (DOCX)
