# Supplementary material for: Prognostic significance of age in 5631 patients with Wilms tumour prospectively registered in International Society of Paediatric Oncology (SIOP) 93-01 and 2001
Source: PLoS One. 2019 Aug 19;14(8):e0221373. doi: 10.1371/journal.pone.0221373 (PMC6699693; doi:10.1371/journal.pone.0221373)
Supplement: S1 Fig — (DOCX) [file pone.0221373.s001.docx]

**S1 Fig. Inclusion flowchart of patients with histologically proven Wilms tumor from the SIOP 93-01 and SIOP 2001 database.**

| Total registered in SIOP 2001 and SIOP 93-01 (1993-2016): ***N=*9288** | | | | | |
| --- | --- | --- | --- | --- | --- |
|  | | | | | |
|  | | | | **Not included:** | |
| Registered as Wilms tumor: ***N=*7842** | | | | Registered as non-Wilms  ***N=*1113** | Category missing or incomplete ***N=*333** |
|  | | | |  |  |
|  | **Not included:** | | |  |  |
| Histologically proven Wilms tumor: ***N=*7262** | Nephroblas-tomatosis / nephrogenic rests ***N=*24** | Non-Wilms histology ***N=*16** | Unknown histology  ***N=*540** |  |  |
|  | | | | | |
| **Inclusion criteria:**  🞎 Treated according to SIOP protocols with pre-operative chemotherapy  🞎 Age ≥6 months and <18 years  🞎 Unilateral disease (stage I-IV) |  |  |  |  |  |
|  | **Not included:** | | | | |
|  | Bilaterals / stage V study patients: ***N=*661**  Other study patients (not treated according to protocol, e.g. primary surgery and/or age <6 months or >18 years): ***N=*817**  Stage missing: ***N=*153** | | | | |
|  |  |  |  |  |  |
| Total included: ***N=*5631** |  |  |  |  |  |
