# Supplementary material for: Prognostic significance of age in 5631 patients with Wilms tumour prospectively registered in International Society of Paediatric Oncology (SIOP) 93-01 and 2001
Source: PLoS One. 2019 Aug 19;14(8):e0221373. doi: 10.1371/journal.pone.0221373 (PMC6699693; doi:10.1371/journal.pone.0221373)
Supplement: S1 Table — (DOCX) [file pone.0221373.s002.docx]

**S1 Table. Previously published literature including age as a variable for outcome in Wilms tumour (WT).**

**Studies with patients treated according to SIOP protocols (with pre-operative chemotherapy):**

| **Reference (year)** | **N** | **Population** | **Outcomes studied** | **Age prognostic?** | **Age categories**  **(years)** | **Univariable analysis**  **(95% CI or *P*-value)** | | **Multivariable analysis**  **(95% CI or *P*-value)** |
| --- | --- | --- | --- | --- | --- | --- | --- | --- |
| Chagtai et al. (2016) [1] | 585 | Stage I-IV in SIOP 2001 registry | EFS | No | *Per year* | *Not described* | | HR 1.01 (1.00-1.01) |
|  |  |  | OS | No | *Per year* | *Not described* | | HR 1.00 (0.99-1.01) |
| Van den Heuvel-Eibrink et al. (2015)[2] | 238 | Stage I-III blastemal-type histology in SIOP 2001 RCT | EFS | No | 2-16 versus <2 | *Not described* | HR 2.21 (1.00–5.93) | |
|  |  |  | OS | No | 2-16 versus <2 | *Not described* | HR 1.61 (0.55–4.78) | |
| Weirich et al. (2004) [3] | 334 | Stage I-V in SIOP9 (registered by GPOH), treated with pre-operative chemotherapy | RFS | **Yes** | 2-16 versus <2 | RR 3.1 (*P=*0.0051) | RR 2.4 (*P=*0.038) | |
|  |  |  | OS | No | 2-16 versus <2 | RR 2.7 (*P=*0.0396) | *Eliminated by backward selection of the proportional hazards model* | |

**Studies with patients treated with primary surgery:**

| **Reference (year)** | **N** | **Population** | **Outcomes studied** | **Age prognostic?** | **Age categories**  **(years)** | **Univariable analysis**  **(95% CI)** | **Multivariable analysis**  **(95% CI)** |
| --- | --- | --- | --- | --- | --- | --- | --- |
| You et al. (2018) [4] | 1924 | Stage I-V in SEER registry | OS | **Yes** | *Per year* | HR 1.045 (1.03-1.06) | HR 1.049 (1.032-1.065) |
| Fernandez et al. (2018)[5] | 535 | Stage III FH WT in AREN0532 | EFS | Univariable only | *Per year* | HR 1.16 (1.08-1.24) | *Age not included in multivariable analysis* |
|  |  |  | OS | No | *Per year* | HR 0.94 (0.77-1.15) |  |
| Saltzman et al. (2018)[6] | 2340 | Stage I-IV FH WT in NCDB | OS | **Yes** | *Per year* | HR 1.106 (1.077-1.136) | HR 1.092 (1.055-1.131) |
| Spreafico et al. (2017) [7] | 453 | Stage I-IV in AIEOP WT 2003 | EFS | No | 2-18 versus <2 | HR 0.72 (0.38-1.35) | HR 1.36 (0.74-2.48) |
|  |  |  | OS | No | 2-18 versus <2 | HR 2.76 (1.07-7.15) | HR 1.63 (0.61-4.34) |
| Jastaniah et al. (2017) [8] | 71 | Stage I-V in a single center in Saudi Arabia | EFS | No | 2-14 versus <2 | HR 0.917 (0.195-4.323) | *Age not included in multivariable analysis* |
|  |  |  | OS | No | 2-14 versus <2 | HR 1.030 (0.293-3.618) |  |
| Irtan et al. (2015) [9] | 635 | Stage I-IV in UKW3 trial | Distant relapse | **Yes, for ≥4 only** | <2  2-4  4-16 | HR 1  HR 1.61 (0.76–3.45)  HR 3.87 (1.95–7.67) | HR 1  HR 1.55 (0.66–3.64)  HR 3.39 (1.56–7.33) |
| Wang et al. (2014) [10] | 1832 | Stage I-V in SEER registry | OS | **Yes** | *Per year* | HR 1.10 (1.05-1.15) | HR 1.09 (1.03-1.14) |
| Provenzi et al. (2014) [11] | 45 | Stage I-V in a single center in Brazil | OS | **Yes** | *Per year* | Not described | HR 1.05 (1.01–1.08) |
| Aronson et al. (2014) [12] | 57 | WT beyond stage I in a single center in South Africa | EFS | No | 0-3  4-7  8-12 | Log rank *P* = 0.956 | *Not performed* |
|  |  |  | OS | No | 0-3  4-7  8-12 | Log rank *P* = 0.797 | *Not performed* |
| Segers et al. (2013) [13] | 331 | Stage I-V WT in the United Kingdom (stage V excluded from survival analysis) | EFS | No | <2  2-4  4-14 | *Not described* | HR 1  HR 1.01 (0.48-2.13)  HR 0.75 (0.34-1.66) |
|  |  |  | OS | No | <2  2-4  4-14 | *Not described* | HR 1  HR 1.15 (0.42–3.15)  HR 0.85 (0.3-2.4) |
| Perotti et al. (2012) [14] | 77 | Stage I-IV WT from AIEOP protocols | RFS | No | 2-18 versus ≤2 | HR 3.776 (0.863-16.520) | HR 2.661 (0.557-12.705) |
| Gutierrez et al. (2010) [15] | 790 | Stage I-V in COG and non-COG centres | OS | No | <1  1-4  5-8  9-18 | *Not described* | HR 1  HR 3.6 4 (0.50–26.67)  HR 3.10 (0.40–23.79)  HR 6.10 (0.74–49.95) |
| Pession et al. (2008) [16] | 555 | Stage I-V in AIEOP registry | OS | **Yes** | 3-14 versus <3 | *Not described* | HR 2.3 (1.4-4.1) |
| Pritchard-Jones et al. (2003) [17] | 242 | Stage I FH WT in UKW2 and UKW3 trial | EFS | **Yes, for ≥4 only** | <2  2-4  4-16 | 93.2%  87.2%  71.3% (Log rank *P* = 0.001) | HR 1  HR 1.53 (0.57-4.11)  HR 3.42 (1.45-8.04) |
|  |  |  | OS | Univariable only | <2  2-4  4-16 | 98.2%  95.0%  87.4% (Log rank *P* = 0.01) | *Not described* |
| Shamberger et al. (1999) [18] | 282 | Stage I-III from NWTS-4 | Local relapse | No | <2  2-4  4-16 | RR 1  RR 0.86 (0.39-1.9)  RR 2.6 (1.4-5.1) | -  -  RR 2.0 (0.95-4) |
| Breslow et al. (1991) [19] | 1466 | Nonmetastatic FH WT in NWTS-3 | Any relapse | **Yes** | <2  2-4  4-16 | 5.4%  9.5%  16.3% (Log rank *P<*0.001) | Regression coefficient 0.15 +/- SE 0.03 |
|  |  |  | Tumor death | **Yes** | <2  2-4  4-16 | 2.1%  4.6%  8.0% (Log rank *P<*0.001) | Regression coefficient 0.15 +/- SE 0.04 |
| Breslow et al. (1985) [20] | 632 | Nonmetastatic WT in NWTS-2 | Any relapse | No | <2  2-4  4-16 | 10.7%  15.8%  22.4% (Log rank *P<*0.001) | “Explained by correlation with other variables” |
|  |  |  | Death | No | <2  2-4  4-16 | 10.2%  13.0%  16.1% (Log rank *P=*0.075) |  |
| D’Angio et al. (1976) [21] | 154 | “Group 1” WT patients in NWTS-1 | DFS at 2 years | **Yes** | <2  ≥2 | 0.89 +/- SE 0.04  0.67 +/- SE 0.06 | *Not performed* |

Legend: SIOP: International Society of Pediatric Oncology, NWTS: National Wilms Tumor Study, COG: Children’s Oncology Group, AIEOP: Associazione Italiana di Ematologia e Oncologia Pediatrica, OS: overall survival, EFS: event-free survival, RFS: relapse-free survival, HR: hazard ratio, RR: relative risk, FH: favourable histology, NCDB: National Cancer Database.

**References:**

1. Chagtai T, Zill C, Dainese L, Wegert J, Savola S, Popov S, et al. Gain of 1q As a Prognostic Biomarker in Wilms Tumors (WTs) Treated With Preoperative Chemotherapy in the International Society of Paediatric Oncology (SIOP) WT 2001 Trial: A SIOP Renal Tumours Biology Consortium Study. Journal of clinical oncology : official journal of the American Society of Clinical Oncology. 2016;34(26):3195-203. Epub 2016/07/20. doi: 10.1200/JCO.2015.66.0001. PubMed PMID: 27432915; PubMed Central PMCID: PMCPMC5505170.

2. van den Heuvel-Eibrink MM, van Tinteren H, Bergeron C, Coulomb-L'Hermine A, de Camargo B, Leuschner I, et al. Outcome of localised blastemal-type Wilms tumour patients treated according to intensified treatment in the SIOP WT 2001 protocol, a report of the SIOP Renal Tumour Study Group (SIOP-RTSG). European journal of cancer (Oxford, England : 1990). 2015;51(4):498-506. Epub 2015/01/17. doi: 10.1016/j.ejca.2014.12.011. PubMed PMID: 25592561.

3. Weirich A, Ludwig R, Graf N, Abel U, Leuschner I, Vujanic GM, et al. Survival in nephroblastoma treated according to the trial and study SIOP-9/GPOH with respect to relapse and morbidity. Annals of oncology : official journal of the European Society for Medical Oncology / ESMO. 2004;15(5):808-20. Epub 2004/04/28. PubMed PMID: 15111352.

4. You H, Yang J, Liu Q, Tang L, Bu Q, Pan Z, et al. The impact of the lymph node density on overall survival in patients with Wilms' tumor: a SEER analysis. Cancer management and research. 2018;10:671-7. Epub 2018/04/20. doi: 10.2147/cmar.s163514. PubMed PMID: 29670401; PubMed Central PMCID: PMCPmc5896671.

5. Fernandez CV, Mullen EA, Chi YY, Ehrlich PF, Perlman EJ, Kalapurakal JA, et al. Outcome and Prognostic Factors in Stage III Favorable-Histology Wilms Tumor: A Report From the Children's Oncology Group Study AREN0532. Journal of clinical oncology : official journal of the American Society of Clinical Oncology. 2018;36(3):254-61. Epub 2017/12/07. doi: 10.1200/jco.2017.73.7999. PubMed PMID: 29211618; PubMed Central PMCID: PMCPmc5773840.

6. Saltzman AF, Carrasco A, Jr., Amini A, Aldrink JH, Dasgupta R, Gow KW, et al. Patterns of lymph node sampling and the impact of lymph node density in favorable histology Wilms tumor: An analysis of the national cancer database. Journal of pediatric urology. 2018;14(2):161.e1-.e8. Epub 2017/11/15. doi: 10.1016/j.jpurol.2017.09.025. PubMed PMID: 29133167.

7. Spreafico F, Biasoni D, Lo Vullo S, Gandola L, D'Angelo P, Terenziani M, et al. Results of the Third AIEOP Cooperative Protocol on Wilms Tumor (TW2003) and Related Considerations. The Journal of urology. 2017;198(5):1138-45. Epub 2017/06/29. doi: 10.1016/j.juro.2017.06.081. PubMed PMID: 28655531.

8. Jastaniah W, Elimam N, Alluhaibi RS, Alharbi AT, Abbas AA, Abrar MB. The prognostic significance of hypertension at diagnosis in children with wilms tumor. Saudi medical journal. 2017;38(3):262-7. Epub 2017/03/03. doi: 10.15537/smj.2017.3.15991. PubMed PMID: 28251221; PubMed Central PMCID: PMCPmc5387902.

9. Irtan S, Jitlal M, Bate J, Powis M, Vujanic G, Kelsey A, et al. Risk factors for local recurrence in Wilms tumour and the potential influence of biopsy - the United Kingdom experience. European journal of cancer (Oxford, England : 1990). 2015;51(2):225-32. Epub 2014/12/04. doi: 10.1016/j.ejca.2014.10.026. PubMed PMID: 25465191.

10. Wang HH, Abern MR, Cost NG, Chu DI, Ross SS, Wiener JS, et al. Use of nephron sparing surgery and impact on survival in children with Wilms tumor: a SEER analysis. The Journal of urology. 2014;192(4):1196-202. Epub 2014/04/17. doi: 10.1016/j.juro.2014.04.003. PubMed PMID: 24735935; PubMed Central PMCID: PMCPmc4194219.

11. Provenzi VO, Rosa RF, Rosa RC, Roehe AV, dos Santos PP, Faulhaber FR, et al. Wilms tumor: experience of a hospital in southern Brazil. Pediatrics international : official journal of the Japan Pediatric Society. 2014;56(4):534-40. Epub 2014/01/23. doi: 10.1111/ped.12295. PubMed PMID: 24447407.

12. Aronson DC, Hadley GP. Age is not a prognostic factor in children with Wilms tumor beyond stage I in Africa. Pediatric blood & cancer. 2014;61(6):987-9. Epub 2014/01/24. doi: 10.1002/pbc.24948. PubMed PMID: 24453084.

13. Segers H, van den Heuvel-Eibrink MM, Williams RD, van Tinteren H, Vujanic G, Pieters R, et al. Gain of 1q is a marker of poor prognosis in Wilms' tumors. Genes, chromosomes & cancer. 2013;52(11):1065-74. Epub 2013/09/17. doi: 10.1002/gcc.22101. PubMed PMID: 24038759.

14. Perotti D, Spreafico F, Torri F, Gamba B, D'Adamo P, Pizzamiglio S, et al. Genomic profiling by whole-genome single nucleotide polymorphism arrays in Wilms tumor and association with relapse. Genes, chromosomes & cancer. 2012;51(7):644-53. Epub 2012/03/13. doi: 10.1002/gcc.21951. PubMed PMID: 22407497.

15. Gutierrez JC, Cheung MC, Zhuge Y, Koniaris LG, Sola JE. Does Children's Oncology Group hospital membership improve survival for patients with neuroblastoma or Wilms tumor? Pediatric blood & cancer. 2010;55(4):621-8. Epub 2010/09/02. doi: 10.1002/pbc.22631. PubMed PMID: 20806361.

16. Pession A, Dama E, Rondelli R, Magnani C, De Rosa M, Locatelli F, et al. Survival of children with cancer in Italy, 1989-98. A report from the hospital based registry of the Italian Association of Paediatric Haematology and Oncology (AIEOP). European journal of cancer (Oxford, England : 1990). 2008;44(9):1282-9. Epub 2008/04/24. doi: 10.1016/j.ejca.2008.03.020. PubMed PMID: 18430564.

17. Pritchard-Jones K, Kelsey A, Vujanic G, Imeson J, Hutton C, Mitchell C. Older age is an adverse prognostic factor in stage I, favorable histology Wilms' tumor treated with vincristine monochemotherapy: a study by the United Kingdom Children's Cancer Study Group, Wilm's Tumor Working Group. Journal of clinical oncology : official journal of the American Society of Clinical Oncology. 2003;21(17):3269-75. Epub 2003/08/30. doi: 10.1200/jco.2003.01.062. PubMed PMID: 12947062.

18. Shamberger RC, Guthrie KA, Ritchey ML, Haase GM, Takashima J, Beckwith JB, et al. Surgery-related factors and local recurrence of Wilms tumor in National Wilms Tumor Study 4. Annals of surgery. 1999;229(2):292-7. Epub 1999/02/19. PubMed PMID: 10024113; PubMed Central PMCID: PMCPmc1191644.

19. Breslow N, Sharples K, Beckwith JB, Takashima J, Kelalis PP, Green DM, et al. Prognostic factors in nonmetastatic, favorable histology Wilms' tumor. Results of the Third National Wilms' Tumor Study. Cancer. 1991;68(11):2345-53. Epub 1991/12/01. PubMed PMID: 1657352.

20. Breslow N, Churchill G, Beckwith JB, Fernbach DJ, Otherson HB, Tefft M, et al. Prognosis for Wilms' tumor patients with nonmetastatic disease at diagnosis--results of the second National Wilms' Tumor Study. Journal of clinical oncology : official journal of the American Society of Clinical Oncology. 1985;3(4):521-31. Epub 1985/04/01. PubMed PMID: 2984345.

21. D'Angio GJ, Evans AE, Breslow N, Beckwith B, Bishop H, Feigl P, et al. The treatment of Wilms' tumor: Results of the national Wilms' tumor study. Cancer. 1976;38(2):633-46. Epub 1976/08/01. PubMed PMID: 184912.
