# Supplementary material for: Prognostic significance of age in 5631 patients with Wilms tumour prospectively registered in International Society of Paediatric Oncology (SIOP) 93-01 and 2001
Source: PLoS One. 2019 Aug 19;14(8):e0221373. doi: 10.1371/journal.pone.0221373 (PMC6699693; doi:10.1371/journal.pone.0221373)
Supplement: S2 Table — (DOCX) [file pone.0221373.s003.docx]

**S2 Table. Prognostic factors for event-free survival (EFS) in patients with Wilms tumor, missing volume imputed (*N=*5631).**

| **Characteristic** | | **Multivariable, age categorized** | | **Multivariable, age linear** | |
| --- | --- | --- | --- | --- | --- |
|  |  | **HR (95% CI)** | **p-value** | **HR (95% CI)** | **p-value** |
| **Sex** | Female | 1 |  | 1 |  |
|  | Male | 0·99 (0·86-1·13) | 0·85 | 0·98 (0·85-1·12) | 0·73 |
| **Age at diagnosis, categorized (years)** | 0-2 | 1 |  |  |  |
|  | 2-4 | 1·34 (1·07-1·67) | 0·0092 |  |  |
|  | 4-10 | 1·80 (1·45-2·22) | <0·0001 |  |  |
|  | 10-18 | 1·74 (1·21-2·49) | 0·0028 |  |  |
| **Age at diagnosis,**  **linear (years)** |  |  |  | 1·06 (1·03-1·08) | <·0001 |
| **Overall stage** | I | 1 |  |  |  |
|  | II | 1·10 (0·90-1·34) | 0·37 | 1·13 (0·93-1·38) | 0·22 |
|  | III | 1·55 (1·27-1·89) | <0·0001 | 1·61 (1·32-1·95) | <0·0001 |
|  | IV | 2·85 (2·37-3·44) | <0·0001 | 2·99 (2·48-3·59) | <0·0001 |
| **Histological risk group** | Intermediate risk | 1 |  |  |  |
|  | High risk: diffuse anaplastic | 2·98 (2·41-3·69) | <0·0001 | 3·18 (2·57-3·92) | <0·0001 |
|  | High risk: blastemal type | 2·24 (1·83-2·74) | <0·0001 | 2·23 (1·82-2·73) | <0·0001 |
|  | Low risk | 0·32 (0·20-0·51) | <0·0001 | 0·33 (0·20-0·53) | <0·0001 |
| **Biopsy** | No | 1 |  |  |  |
|  | Yes | 1·05 (0·87-1·28) | 0·601 | 1·02 (0·84-1·24) | 0·86 |
| **Volume at surgery** | ≤500 ml | 1 |  |  |  |
|  | >500 ml | 1·99 (1·66-2·40) | <0·0001 | 1·92 (1·60-2·30) | <0·0001 |
