# Supplementary material for: Prognostic significance of age in 5631 patients with Wilms tumour prospectively registered in International Society of Paediatric Oncology (SIOP) 93-01 and 2001
Source: PLoS One. 2019 Aug 19;14(8):e0221373. doi: 10.1371/journal.pone.0221373 (PMC6699693; doi:10.1371/journal.pone.0221373)
Supplement: S3 Table — (DOCX) [file pone.0221373.s004.docx]

**S3 Table. Prognostic factors for event-free survival (EFS) in patients with Wilms tumor, SIOP 2001 only (*N=3132*).**

| **Characteristic** | | **Multivariable, age categorized** | | **Multivariable, age linear** | |
| --- | --- | --- | --- | --- | --- |
|  |  | **HR (95% CI)** | **p-value** | **HR (95% CI)** | **p-value** |
| **Sex** | Female | 1 |  | 1 |  |
|  | Male | 1.05 | 0.62 | 1.04 |  |
| **Age at diagnosis, categorized (years)** | 0-2 | 1 |  |  |  |
|  | 2-4 | 1.32 | 0.07 |  |  |
|  | 4-10 | 1.82 | <0.0001 |  |  |
|  | 10-18 | 1.85 | 0.01 |  |  |
| **Age at diagnosis,**  **linear (years)** |  |  |  | 1.07 | <0.0001 |
| **Overall stage** | I | 1 |  | 1 |  |
|  | II | 1.23 | 0.13 | 1.26 | 0.09 |
|  | III | 1.48 | 0.004 | 1.51 | 0.003 |
|  | IV | 2.87 | <0.0001 | 3.01 | <0.0001 |
| **Histological risk group** | Intermediate risk | 1 |  | 1 |  |
|  | High risk: diffuse anaplastic | 3.24 | <0.0001 | 3.42 | <0.0001 |
|  | High risk: blastemal type | 1.60 | 0.002 | 1.58 | 0.003 |
|  | Low risk | 0.25 | 0.0001 | 0.26 | 0.0002 |
| **Biopsy** | No | 1 |  | 1 |  |
|  | Yes | 1.19 | 0.16 | 1.14 | 0.32 |
| **Volume at surgery** | ≤500 ml | 1 |  | 1 |  |
|  | >500 ml | 1.79 | <0.0001 | 1.73 | <0.0001 |
