# Supplementary material for: Prognostic significance of age in 5631 patients with Wilms tumour prospectively registered in International Society of Paediatric Oncology (SIOP) 93-01 and 2001
Source: PLoS One. 2019 Aug 19;14(8):e0221373. doi: 10.1371/journal.pone.0221373 (PMC6699693; doi:10.1371/journal.pone.0221373)
Supplement: S4 Table — (DOCX) [file pone.0221373.s005.docx]

**S4 Table. Prognostic factors for overall survival (OS) in patients with Wilms tumour, missing volume imputed (*N=*5631).**

| **Characteristic** | | **Multivariable, age categorized** | | **Multivariable, age linear** | |
| --- | --- | --- | --- | --- | --- |
|  |  | **HR (95% CI)** | **p-value** | **HR (95% CI)** | **p-value** |
| **Sex** | Female | 1 |  | 1 |  |
|  | Male | 0·84 (0·68-1·04) | 0·11 | 0·83 (0·68-1·03) | 0·09 |
| **Age at diagnosis, categorized (years)** | 0-2 | 1 |  |  |  |
|  | 2-4 | 1·17 (0·81-1·69) | 0·40 |  |  |
|  | 4-10 | 1·52 (1·07-2·17) | 0·02 |  |  |
|  | 10-18 | 1·75 (1·04-2·96) | 0·03 |  |  |
| **Age at diagnosis,**  **linear (years)** |  |  |  | 1·04 (1·00-1·07) | 0·05 |
| **Overall stage** | I | 1 |  | 1 |  |
|  | II | 1·56 (1·09-2·23) | 0·01 | 1·60 (1·12-2·28) | 0·01 |
|  | III | 3·13 (2·26-4·33) | <0·0001 | 3·22 (2·33-4·46) | <0·0001 |
|  | IV | 6·98 (5·13-9·50) | <0·0001 | 7·28 (5·36-9·88) | <0·0001 |
| **Histological risk group** | Intermediate risk | 1 |  | 1 |  |
|  | High risk: diffuse anaplastic | 6·80 (5·16-8·96) | <0·0001 | 7·12 (5·43-9·32) | <0·0001 |
|  | High risk: blastemal type | 4·44 (3·37-5·85) | <0·0001 | 4·51 (3·42-5·94) | <0·0001 |
|  | Low risk | 0·47 (0·25-0·89) | 0·02006 | 0·48 (0·25-0·92) | 0·03 |
| **Biopsy** | No | 1 |  | 1 |  |
|  | Yes | 1·00 (0·75-1·34) | 0·98 | 1·01 (0·76-1·35) | 0·95 |
| **Volume at surgery** | ≤500 ml | 1 |  | 1 |  |
|  | >500 ml | 2·19 (1·69-2·84) | <0·0001 | 2·17 (1·67-2·81) | <0·0001 |
