# Supplementary material for: Prognostic significance of age in 5631 patients with Wilms tumour prospectively registered in International Society of Paediatric Oncology (SIOP) 93-01 and 2001
Source: PLoS One. 2019 Aug 19;14(8):e0221373. doi: 10.1371/journal.pone.0221373 (PMC6699693; doi:10.1371/journal.pone.0221373)
Supplement: S5 Table — (DOCX) [file pone.0221373.s006.docx]

**S5 Table. Prognostic factors for overall survival (OS) in patients with Wilms tumour, SIOP 2001 only (*N=3132*).**

| **Characteristic** | | **Multivariable, age categorized** | | **Multivariable, age linear** | |
| --- | --- | --- | --- | --- | --- |
|  |  | **HR (95% CI)** | **p-value** | **HR (95% CI)** | **p-value** |
| **Sex** | Female | 1 |  | 1 |  |
|  | Male | 0.83 | 0.22 | 0.82 | 0.2 |
| **Age at diagnosis, categorized (years)** | 0-2 | 1 |  |  |  |
|  | 2-4 | 1.08 | 0.78 |  |  |
|  | 4-10 | 1.58 | 0.08 |  |  |
|  | 10-18 | 1.52 | 0.29 |  |  |
| **Age at diagnosis,**  **linear (years)** |  |  |  | 1.05 | 0.09 |
| **Overall stage** | I | 1 |  | 1 |  |
|  | II | 1.93 | 0.02 | 1.96 | 0.01 |
|  | III | 3.49 | <0.0001 | 3.52 | <0.0001 |
|  | IV | 9.87 | <0.0001 | 10.27 | <0.0001 |
| **Histological risk group** | Intermediate risk | 1 |  | 1 |  |
|  | High risk: diffuse anaplastic | 10.06 | <0.0001 | 10.8 | <0.0001 |
|  | High risk: blastemal type | 3.56 | <0.0001 | 3.57 | <0.0001 |
|  | Low risk | 0.51 | 0.15 | 0.53 | 0.18 |
| **Biopsy** | No | 1 |  | 1 |  |
|  | Yes | 1.13 | 0.53 | 1.11 | 0.6 |
| **Volume at surgery** | ≤500 ml | 1 |  | 1 |  |
|  | >500 ml | 2.13 | <0.0001 | 2.14 | <0.0001 |
