# Supplementary material for: Prognostic significance of age in 5631 patients with Wilms tumour prospectively registered in International Society of Paediatric Oncology (SIOP) 93-01 and 2001
Source: PLoS One. 2019 Aug 19;14(8):e0221373. doi: 10.1371/journal.pone.0221373 (PMC6699693; doi:10.1371/journal.pone.0221373)
Supplement: S6 Table — (DOCX) [file pone.0221373.s007.docx]

| **Reference (year)** | **N** | **Population** | **Outcomes studied** | **Factors included in multivariable analysis (95% CI or *P*-value) - *significant factors in bold*** | | | | |
| --- | --- | --- | --- | --- | --- | --- | --- | --- |
|  |  |  |  | **Age** | **1q gain** | **1p loss/LOH** | **16q loss/LOH** | **Other factors:** |
| Perotti et al. (2012)[1] | 77 | Stage I-IV WT in AIEOP registry | RFS | HR 2.66 (0.56-12.71) | **HR 6.11 (1.70-21.96)** | *Not included* | *Not included* | Stage, presence of diffuse anaplasia |
| Segers et al. (2013)[2] | 331 | Stage I-V WT in the United Kingdom (stage V excluded from survival analysis) | EFS | 0-2: HR 1  2-4: HR 1.01 (0.48-2.13)  >4: HR 0.75 (0.34-1.66) | **HR 2.45 (1.17-5.15)** | HR 0.37 (0.1-1.37) | HR 1.83 (0.8-4.16) | Stage, 11q-. 16q-, 22-, cx, 14q-, 4q-, 8+, 10+, 12+ |
|  |  |  | OS | 0-2: HR 1  2-4: HR 1.15 (0.42-3.25)  >4: HR 0.85 (0.3-2.4) | **HR 4.28 (1.59-11.53)** | HR 0.23 (0.05-1.1) | HR 1.55 (0.56-4.33) |  |
| Chagtai et al. (2016)[3] | 586 | Stage I to IV WT in SIOP 2001 | EFS | HR 1.01/year (1-1.01) | **HR 1.98 (1.27-3.07)** | HR 0.98 (0.5-1.91) | HR 1.14 (0.68-1.91) | Gender, **stage, histology (high vs. intermediate risk)** |
|  |  |  | OS | HR 1/year (0.99-1.01) | HR 1.61 (0.83-3.15) | HR 0.67 (0.23-1.89) | HR 1.37 (0.67-2.83) |  |
| Gratias et al. (2016)[4] | 1114 | Stage I-IV non-anaplastic WT, exluding patients with stage I very-low risk WT in NWTS | EFS | Not included in MVA, however: patients with 1q gain were found to be older (median age 51.5 months) than those without 1q gain (median age 36.5 months), *P*=0.001 | **RR 2.4 (*P*<0.001)** | Not significant in MVA; however: in the group of patients lacking 1q gain; 1p and/or 16q loss seems to be associated with EFS (EFS 84% vs. 91%, *P*=0.03). | | **Stage** |
|  |  |  | OS |  | RR 3.08 (*P*=0.067*)** |  |  |  |

**S6 Table. Previously published studies including 1q gain in multivariable analysis.**

Legend: WT: Wilms tumour, SIOP: International Society of Pediatric Oncology, NWTS: National Wilms Tumor Study, AIEOP: Associazione Italiana di Ematologia e Oncologia Pediatrica, OS: overall survival, EFS: event-free survival, RFS: relapse-free survival, HR: hazard ratio, RR: relative risk.

**References:**

1. Perotti D, Spreafico F, Torri F, Gamba B, D'Adamo P, Pizzamiglio S, et al. Genomic profiling by whole-genome single nucleotide polymorphism arrays in Wilms tumor and association with relapse. Genes, chromosomes & cancer. 2012;51(7):644-53. Epub 2012/03/13. doi: 10.1002/gcc.21951. PubMed PMID: 22407497.

2. Segers H, van den Heuvel-Eibrink MM, Williams RD, van Tinteren H, Vujanic G, Pieters R, et al. Gain of 1q is a marker of poor prognosis in Wilms' tumors. Genes, chromosomes & cancer. 2013;52(11):1065-74. Epub 2013/09/17. doi: 10.1002/gcc.22101. PubMed PMID: 24038759.

3. Chagtai T, Zill C, Dainese L, Wegert J, Savola S, Popov S, et al. Gain of 1q As a Prognostic Biomarker in Wilms Tumors (WTs) Treated With Preoperative Chemotherapy in the International Society of Paediatric Oncology (SIOP) WT 2001 Trial: A SIOP Renal Tumours Biology Consortium Study. Journal of clinical oncology : official journal of the American Society of Clinical Oncology. 2016;34(26):3195-203. Epub 2016/07/20. doi: 10.1200/JCO.2015.66.0001. PubMed PMID: 27432915; PubMed Central PMCID: PMCPMC5505170.

4. Gratias EJ, Dome JS, Jennings LJ, Chi YY, Tian J, Anderson J, et al. Association of Chromosome 1q Gain With Inferior Survival in Favorable-Histology Wilms Tumor: A Report From the Children's Oncology Group. Journal of clinical oncology : official journal of the American Society of Clinical Oncology. 2016;34(26):3189-94. Epub 2016/07/13. doi: 10.1200/JCO.2015.66.1140. PubMed PMID: 27400937; PubMed Central PMCID: PMCPMC5012705 online at <www.jco.org>. Author contributions are found at the end of this article.
